# Scientific standards and MIAPEs in plant proteomics research and publications

**DOI:** 10.3389/fpls.2015.00473

**Published:** 2015-06-30

**Authors:** Jesús V. Jorrín Novo

**Affiliations:** Agroforestry and Plant Biochemistry and Proteomics Research Group, Department of Biochemistry and Molecular Biology- ETSIAM, University of Cordoba-CeiA3Córdoba, Spain

**Keywords:** plant proteomics, scientific standards, MIAPE, scientific publications, comparative proteomics

“*I suspect that the authors are capable of doing a much better job of preparing a scientific Ms., and I wish that they had applied more effort with this one. If the writing and Ms. preparation are this sloppy and amateurish, can the research be trusted? The authors simply list quantitative results, and make some broad, generalized, pedestrian comments. Hardly a Discussion*.”

(Anonymous referee)

In this opinion paper it is my intent to briefly discuss some key issues related to scientific standards in plant proteomics research and the “Minimal Information About a Proteomics Experiment” (MIAPEs) requested for derived publications. It is mainly aimed at beginners rather than scientists who have established a long trajectory and experience within the field, trying to rationally connect proteomics and plant biology. The content was presented at the “1st INPPO World Congress on Plant Proteomics: Methodology to Biology,” September 2014, and has been discussed in reviews published by the author, with the most recent referenced herein (Jorrin Novo et al., 2009; Jorrin Novo et al., 2014, 2015; Valledor and Jorrin, 2011). As an opinion paper it should be, what else!¡, subjected to comments, disagreements, or criticisms, but at the very least open discussion. It reflects 12 years of active research, as an author (who has had some experience with rejected manuscripts), a reviewer, and an editor who has handled around 400 manuscripts (about 50% of which were ultimately accepted).

MS-based Proteomics, as an analytical tool, has developed to an unanticipated level in a very short period of time. Even so, its potential remains far from being fully exploited especially as a component of plant biology in comparison with other organisms (i.e., humans, yeast, bacteria). Some areas (PTMs, interactomics) or techniques (targeted, arrays, imaging) are minimally represented in the current plant literature, while others (protein trafficking, degradation, protein function at the –omics level) remain absent or anecdotal. Despite being a powerful technique, it has limitations (quantitation, orphan organisms). As my friend Juan Pablo Albar (recently deceased) used to say, “Jesus, real proteomics is only possible when studying organisms with a sequenced genome as we should pretend to identify gene products.” It is not a panacea, or a miracle. By itself it is almost impossible to unravel biological processes. Results must be validated, and contrasted with those obtained by using biochemical, molecular (classical, other –omics), or cellular biology approaches. We now appreciate that the protein world is much more complex from a structural and functional point of view than ever imagined. It is increasingly clear that in its present state, proteomics is mostly descriptive and to a great extent speculative. While description is valuable by itself, it is not always adequate to support biological speculations or support speculative conclusions. While this opinion might be considered controversial by some, it is shared by others and is well presented in the last review by Paola Picotti (Boersema et al., 2015). Because of this evolution in the nature of (plant) proteomics, we have chosen to present a philosophical rather than data-based contribution.

For those with limited prior experience, but with a well-designed biological project, it is important to remember that proteomic analysis is much more than just sending samples to a Proteomics Service (you should not pretend that mass spectrometists are knowledgeable about plant biology), then blindly accepting the results, and preparing a more or less confident protein identification and quantification table. Only if one understands both the experimental system and the proteomics techniques applied can we understand the results well enough to speculate about how, why, and what insight the results provide? Proteomics has innate limitations which must be taken into account; data must be critically evaluated, correctly validated and interpreted, and finally, submitted manuscripts should fit into the general scientific and particular proteomics standards or MIAPEs (Minimum Information About a Proteomics Experiment). Such a MIAPEs have been translated to a number of documents elaborated by the Proteomics Standard Initiative within the Human Proteomics Organization (HUPO; http://www.psidev.info/node/91); they are related to “community standards for data representation in proteomics to facilitate data comparison, exchange and verification” (Orchard et al., 2003; http://www.psidev.info/), making reference to each of the steps in a standard proteomics workflow (gel electrophoresis, gel informatics, MS general, MS informatics, MS quantitation, column chromatography, capillary electrophoresis, molecular interaction). These standards are requested by the four top-ranking journals in the field (by year of appearance, Proteomics, Molecular, and Cellular Proteomics, Journal of Proteome Research, and Journal of Proteomics), and briefly summarized in the corresponding journal instruction to authors. Other journals that publish proteomics data are typically more concerned with the biological contribution than a complete description of the methods used, and thus do not highlight these standards. However it behooves us all in the plant proteomics community (researchers, reviewers, editors) to strictly adhere to both these standards and MIAPEs.

When considering a manuscript for publication, apart from formal aspects (English edition and format requirements as indicated in the instruction to authors), the failure to meet general scientific standards is the most obvious and immediate reason for rejection. It is important to keep these standards in mind throughout the processes of planning, conducting, interpreting, and describing an experiment: (i) Experimental design (number of experiments, biological or analytical replicates, sample size, sample homogeneity); (ii) Method optimization and validation (the employed techniques must be validated from an analytical point of view, and specificity, precision, accuracy, dynamic range, limit of detection, limit of quantitation, should be known); (iii) Analysis of the data and statistics); and (iv) Interpretation of the data (taking into account the experimental design, the employed methods, and the statistical analysis). Reproducibility and bias minimization, as well as validity of the data from a biological point of view (the extent to which similar findings are reported using other experimental systems and/or approaches) are also key issues. The proteome is dynamic, even for clonal and synchronized cells, and because of this the mean coefficient of variance of a proteome is quite variable. Furthermore, it is crucial to remember that the proteomic results we describe and interpret from a biological point of view are but a single fixed photograph of a whole movie. We cannot pretend to fathom very complex biological processes from the results of a single experiment, even if we have resolved and identified thousands of protein species. Typically, the plant samples being analyzed include a complex mixture of tissues and cell types, each of which has its own protein signature and not all of which respond identically to the experimental variables.

The performed work can be translated to an acceptable manuscript if: (i) The main contributions to the experimental system or biological process are clearly presented and summarized in the abstract and introduction, ensuring its understanding (going beyond just a description of the proteome as far as possible without pretending to review the covered topic); (ii) proper terminology is correctly used; (iii) the methods section is written such as it ensures the repetition of the experiments by any who, anywhere; (iv) results, original in preference to very elaborately analyzed data, are presented; and (v) the discussion section does not contain unwarranted speculations but rather conclusions and hypotheses supported by the data presented. In the table accompanying this opinion paper (Table 1) is summarized the major causes of rejection of a submitted manuscripts or at least the main criticisms based on the author's experience as editor and/or author. One specific issue deserves some emphasis; the use of scientific terms. It is critical that we are all (authors, reviewers, editors, and readers) considering the same thing! Scientific terminology must be very precise and unambiguous, although it is also true that archaic can be productively adapted and properly interpreted in the context of contemporary techniques. The literal translation of specific terms from genomics to proteomics can also generate confusion. The scientific community should discuss and agree on that, and the creation of a nomenclature committee is a need. For example, the use of “protein species” or “protein forms” rather than just “proteins,” as previously proposed (Jorrin et al., 2006; Schlutter et al., 2009; Smith et al., 2013). As far as possible it should be clearly stated which gene product is referred to, and whether the protein species corresponds to a multigene family, isogene, or allelic variant. In the case of orphan organisms whose genome is not sequenced reference to the orthologs should be made. Also, based on proteomics experiments we can only describe differences in protein species abundance. Terms such as differences in protein expression (in fact the genes are expressed), up or down-regulation, induction, repression, must be avoided. Up or down gene expression is just one of the possible mechanisms explaining differences in protein abundance.

**Table 1:** **Most general comments and criticisms to plant proteomics manuscripts posted by different reviewers as recorded by the author in his task as editor**.

**Issue**	**Comments**
Terminology	Commented in the main text.
Experimental design	The experimental design must be provided and must include details of the number of biological and analytical replicates. A picture of the experimental system should be included as supplementary material. The biological or technical replicate must be clearly defined (number of leaves/roots/seeds… coming from X number of plants, number of cells,… per replicate, fresh or even better dry weight).
Protein quantification	It should be provided, with the caveat that typical methods overestimate the amount of protein in a crude extract and the value depends on the standard protein used. Units correspond to equivalents of the standard protein.
Comparative proteomics (i.e., 2-DE based)	A table containing columns devoted to: (i) protein yield (per dry weight bases); (ii) number of spots (mean and SD); (iii) Number of variable spots, taking as reference one of the sample; (iv) qualitative differences (newly appeared/disappeared); (v) quantitative differences (up/down accumulated), should be included. Indicate when a spot is considered variable? (consistency among replicates; statistics; ratios). Comment on coefficient of variance for the samples.
Statistics	Data from all the samples must be considered as a whole and because of that, multivariant analysis of the variance should be used (Valledor and Jorrin, 2011). The combination of both univariate and multivariate approaches provides a comprehensive overview of the data, with single protein studies and multiprotein trends, maximizing the information obtained from most of the datasets.
Protein identification	We should go beyond just blindly accepting the data produced by the used software packages. Identification of proteins from organisms with unknown genome sequence will be accepted only if MS/MS-derived peptide sequence data have been used for database searching or BLAST analysis. The score for the highest ranked hit to a homologous, orthologous, or paralogous protein should be indicated. Present the identification table in a proper way, indicating accession number and organisms, and putative existence of isoforms (products of different genes), cellular locations, Exp/Theor. Mr/pI, score (protein score and peptide score), number of peptides, covered sequence, and false discovered rate. Peptide sequence and charge should be included as supplementary material. Organize the table according to the function. From a proteomics perspective, protein species identification should be discussed in terms of the same or different gene products (protein species, isoforms, allelic variants), PTMs (variants with different pI or Mr). While possible, DNA, EST or protein sequences of the own or closest organism must be used, even if the number of available sequences is low (Romero-Rodriguez et al., 2014).
Discussion	The discussion section must explore the significance of the results of the work and the contribution to the biology/proteomics field. We should not pretend to review the identified proteins. Apart from that it is important to discuss cases where different spots matched the same protein function.
To be the first in reporting	This formula does not necessarily work or it is irrelevant. The work could be of potential interest considering the experimental system, X, mostly unknown. However the work and corresponding manuscript does not fit the standards required for a proteomics publication. A deeper proteomics analysis and data validation is necessary in order to conclude from a biological point of view. Otherwise it is only descriptive and speculative. The proteome description in this type of orphan organisms is of value but the number of proteins identified is quite low.

*These comments mostly apply to comparative proteomics papers that represent the higher percentage of the submitted manuscripts. The table could be completed with the MIAPE standards (http://www.psidev.info/node/91)*.

Finally, I advise being modest and humble when dealing with proteomics research. Contemporary MS-based proteomics methods can generate huge datasets in a relatively short period of time. This is quite different from even the recent past. For my thesis I spent 4 years working with just one protein, the enzyme phenylalanine ammonia-lyase. Final analyses will depend on the comparisons made. In a single experiment, the best results we can imagine will include less than 10% of the total proteome. Because of this, I suggest replacing “proteome” with “extractome” in most instances. This is based upon the following considerations:

- How many proteins are extracted and solubilized? If so, unmodified?
- How many proteins are lost during separation (1-DE, 2-DE, LC, nLC)?
- How many proteins are visualized or provide signals during separation?
- How many peptides are generated and how many are lost during the digestion step?
- Are all the peptides getting to the mass detector? Do they provide signal (m/z)? Are they fragmented (MS^n^)?
- Do the MS data lead to protein identification, depending on the used algorithms and databases?

While there are many other points that should be described/discussed/argued about in terms of the future of plant proteomics. Like the proteins we address, the field itself if dynamic and should be continuously evolving. Other specific points have not been incorporated due to the length restrictions by the journal for an opinion paper.

There is life beyond descriptive proteomics, and it is increasingly important that we consider biological context. If the results obtained do not fit our hypotheses (or preconceptions?) or fail to provide satisfactory answers to our research questions, then we must avoid the temptation to “tread water,” and move on to application of additional approaches be they experimental or computational.

## Conflict of interest statement

The author declares that the research was conducted in the absence of any commercial or financial relationships that could be construed as a potential conflict of interest.
